# Nutritional Counseling Is Independently Associated with Greater Knowledge of Drug–Food Interactions in Patients with Type 2 Diabetes

**DOI:** 10.3390/nu18050742

**Published:** 2026-02-26

**Authors:** Joanna Korbela, Agnieszka Białek

**Affiliations:** 1 School of Health and Medical Sciences, VIZJA University, Okopowa 59, 01-043 Warsaw, Poland; korbej4317425_aeh@students.vizja.pl; 2 The Kielanowski Institute of Animal Physiology and Nutrition, Polish Academy of Sciences, Instytucka 3, 05-110 Jabłonna, Poland

**Keywords:** GLP-1 receptor agonists, drug-food interactions (DFIs), patient knowledge, type 2 diabetes, nutritional support, diabetes education

## Abstract

**Background:** Type 2 diabetes mellitus (T2DM) is commonly managed with complex pharmacotherapy combined with dietary modification, which increases the risk of clinically relevant drug–food interactions (DFIs). Despite their potential impact on treatment efficacy and safety, patient knowledge of DFIs—particularly in the context of modern therapies such as glucagon-like peptide-1 receptor agonists (GLP-1 RAs)—remains insufficiently explored. **Methods:** This cross-sectional study assessed knowledge of DFIs among 103 adults with T2DM using a self-administered, expert-validated questionnaire. Data on sociodemographic characteristics, clinical variables, anti-diabetic therapy (including GLP-1 RAs), sources of education, and attendance at dietary consultations were collected. Knowledge scores were calculated based on correct responses and categorized into tertiles (low, moderate, high). Associations were analyzed using non-parametric tests. Multivariable logistic regression was performed to identify independent predictors of moderate-to-high DFI knowledge. **Results:** Substantial gaps in DFI knowledge were identified, particularly regarding interactions involving dietary fiber, dairy products, grapefruit juice, and nutrient deficiencies associated with long-term pharmacotherapy. Knowledge level was not significantly associated with age, educational attainment, diabetes duration, or GLP-1 RA use. Female sex was associated with higher knowledge in univariate analysis (*p* = 0.026); however, this association did not remain significant in the multivariable regression model. Attendance at at least one dietary consultation in the previous year was significantly associated with higher knowledge levels (*p* = 0.041) and remained an independent predictor in multivariable analysis (OR = 2.31; 95% CI: 1.04–5.15; *p* = 0.039). Most participants reported not receiving prior education on DFIs, while expressing a strong need for more frequent counseling. **Conclusions:** Patients with T2DM demonstrate insufficient knowledge of clinically relevant DFIs, including selected issues related to GLP-1 RA therapy. Attendance at structured dietary consultations was independently associated with higher levels of DFI knowledge; however, the directionality and causality of this relationship cannot be established. Given the cross-sectional design and the assessment of knowledge rather than behavioral or clinical outcomes, these findings should be interpreted as hypothesis-generating. Further longitudinal and interventional studies are required to determine whether improved DFI knowledge translates into meaningful changes in dietary behavior, treatment adherence, or metabolic outcomes.

## 1. Introduction

Type 2 Diabetes Mellitus (T2DM) is a major global public health challenge affecting hundreds of millions of individuals worldwide. Its chronic and progressive nature, driven by insulin resistance and impaired β-cell function, requires long-term, multifaceted management strategies. Effective treatment encompasses glycemic control, management of comorbidities, and lifestyle modification, with medical nutrition therapy constituting a cornerstone of care [1,2]. As T2DM frequently coexists with obesity, hypertension, and dyslipidemia, patients are commonly treated with complex multidrug regimens aimed at preventing microvascular and macrovascular complications, including major adverse cardiovascular events (MACE) [3].

The widespread use of polypharmacy in T2DM substantially increases the risk of drug-food interactions (DFIs). DFIs occur when food components or dietary patterns alter drug pharmacokinetics and/or pharmacodynamics, or when medications interfere with nutrient absorption or metabolism [4,5]. Clinically relevant DFIs may reduce therapeutic efficacy, impair disease control, or increase the risk of adverse effects, thereby compromising patient safety and treatment adherence. Well-established examples include CYP3A4 inhibition by grapefruit juice and the interaction between dietary vitamin K intake and warfarin therapy [6]. Given the lifelong nature of pharmacological treatment in T2DM, even low-grade but persistent DFIs may have cumulative clinical consequences, underscoring the importance of patient education in this area.

In recent years, the therapeutic landscape for T2DM and obesity has been transformed by the introduction of novel, highly effective drug classes, notably the glucagon-like peptide-1 receptor agonists (GLP-1 RAs). These agents, which act as incretin mimetics to enhance glucose-dependent insulin secretion, suppress glucagon release, and promote satiety via central mechanisms, have demonstrated potent effects on glycemic control and, critically, robust outcomes in terms of sustainable weight reduction and substantial cardiovascular risk mitigation [7,8]. The inclusion of GLP-1 RAs into standard care protocols, often in combination with agents like metformin (as observed in over 85% of patients in the current study cohort), introduces new considerations for optimal drug administration and nutritional support. A key pharmacological action of GLP-1 RAs is the slowing of gastric emptying, which directly impacts the absorption rate of both food and concurrently administered oral medications [9].

Effective management of patients on GLP-1 RA therapy therefore requires targeted nutritional counseling to maximize therapeutic benefits and, equally important, to mitigate common gastrointestinal side effects (e.g., nausea, vomiting, constipation, or diarrhea) that are predominantly managed through specific dietary adjustments [10]. This critical need for precise nutritional guidance highlights the direct link between dietary knowledge and successful GLP-1 RA treatment outcomes.

Despite the clinical significance of DFIs, studies consistently indicate a concerning lack of adequate patient knowledge regarding the proper administration of anti-diabetic medications and the potential influence of diet [11]. The preliminary data supporting this article suggests that a majority of patients (73%) recognize the fundamental importance of education on anti-diabetic drug-food interactions, yet critical knowledge gaps persist concerning the impact of common dietary components such as high-fiber products, dairy, and fruit juices (e.g., grapefruit) on medication efficacy and safety [12]. Given the increasing prevalence of GLP-1 RA therapy, it is imperative to ascertain whether patients possess the specific knowledge required to safely and effectively navigate the dietary complexities associated with this particular drug class.

Despite the growing body of literature addressing drug–drug interactions and general DFIs in chronic diseases, data specifically evaluating patient awareness of DFIs in the context of GLP-1 RA therapy remain scarce. Most available studies have focused either on pharmacokinetic interactions at a mechanistic level or on general patient knowledge of medication use, without differentiating between specific anti-diabetic drug classes or accounting for their unique nutritional implications. Importantly, GLP-1 RAs represent a distinct therapeutic category in which pharmacological efficacy, tolerability, and long-term adherence are closely intertwined with dietary behaviors and nutritional support. Through their well-established effect on gastric emptying, GLP-1 RAs may alter the absorption kinetics of concomitantly administered oral medications and interact with meal composition, timing, and nutrient content (e.g., fiber or fat intake) [13]. In addition, emerging evidence suggests that patients treated with GLP-1 RAs frequently fail to meet dietary recommendations, including adequate fiber intake and overall dietary quality, despite pharmacologically induced appetite suppression [14,15]. Recent narrative reviews and advisory statements further emphasize that nutritional guidance is a critical yet under-researched component of GLP-1-based therapies, particularly in relation to managing gastrointestinal adverse effects and optimizing long-term cardiometabolic outcomes [16]. However, existing research has largely overlooked whether patients receiving GLP-1 RA possess sufficient knowledge to appropriately adapt their dietary habits and medication use to these pharmacological characteristics. Consequently, a clear gap exists in the literature regarding the assessment of patient knowledge of DFIs specifically relevant to GLP-1 RA therapy and its nutritional support. Addressing this gap is essential, as insufficient awareness may compromise treatment effectiveness, exacerbate gastrointestinal side effects, reduce adherence, and ultimately limit the cardiometabolic benefits associated with GLP-1-based therapies.

Therefore, the primary objective of this study was to evaluate the level of knowledge regarding DFIs among patients with T2DM, with a specific focus on the subset of patients undergoing therapy with GLP-1 RA. By identifying specific deficits and areas of confusion, the findings aim to provide evidence-based recommendations for healthcare professionals, including physicians, pharmacists, and dietitian, to design and implement targeted educational programs. Ultimately, this work seeks to enhance nutritional support of GLP-1 RA therapy, thereby optimizing patient safety, adherence, and long-term clinical outcomes in the comprehensive management of T2DM.

## 2. Materials and Methods

### 2.1. Experimental Approach

This was a cross-sectional, questionnaire-based study conducted among adult patients with T2DM. Data were collected between April and June 2025 using a structured questionnaire administered in both paper-based and online formats. The study aimed to assess patient knowledge regarding DFIs and to identify factors associated with higher knowledge, with a particular focus on nutritional counseling and the use of GLP-1 RA.

The study protocol and methodology adhered to the Declaration of Helsinki and were reviewed and approved by the Bioethics Committee of VIZJA University (approval number: 04/04/2025). Participation was voluntary and anonymous. At the beginning of the questionnaire, participants were informed about the study aims, the anonymous nature of data collection, and their right to withdraw at any time without providing a reason. Completion of the questionnaire was considered as implied informed consent, in accordance with standard practice for anonymous questionnaire-based research and as approved by the Bioethics Committee.

### 2.2. Study Participants and Recruitment

The study group consisted of 103 adult patients diagnosed with T2DM. A convenience sampling method was employed to recruit participants from various clinical and non-clinical settings, ensuring a broad representation of the patient population. Recruitment was carried out in: (i) inpatient and outpatient departments of a general hospital (e.g., Diabetes Clinic), (ii) primary care facilities (POZ) and (iii) online platforms and patient communities specializing in diabetes management. This recruitment approach was intended to facilitate access to a heterogeneous group of patients but may also have introduced selection bias related to health-seeking behaviors and sociodemographic characteristics. Inclusion criteria were: (i) confirmed diagnosis of T2DM, (ii) age > 18 years, and (iii) ability to provide informed consent and complete the questionnaire independently. Exclusion criteria included cognitive impairment or any condition preventing comprehension of the questionnaire. No a priori sample size calculation was performed, as the study was exploratory in nature. Data regarding demographic characteristics (e.g., age, gender, education level), clinical factors (e.g., duration of T2DM, comorbidities), and current pharmacological treatment (including the use of GLP-1 RA) were collected to characterize the study group. Female sex was associated with higher knowledge in univariate analysis (*p* = 0.026); however, this association did not remain significant in the multivariable regression model.

### 2.3. Data Collection Tool

Data were collected using an original, self-developed survey questionnaire (Supplementary Materials) specifically designed for this study, consisting exclusively of closed-ended questions. The questionnaire was structured into three main parts:

Part I: Demographic and clinical data—questions pertaining to socio-economic status, diabetes duration, coexisting diseases, and medications used (including metformin, insulin, sulfonylureas, and GLP-1 RAs).

Part II: Assessment of DFI knowledge—this section included questions assessing knowledge of clinically relevant DFIs in T2DM therapy, such as appropriate timing of anti-diabetic drug administration relative to meals, consequences of alcohol consumption during metformin therapy, interactions between grapefruit juice and selected medications (e.g., via CYP3A4 inhibition), and principles related to the mechanism of action and nutritional support of GLP-1 RAs.

Part III: Assessment of nutritional knowledge and education needs—questions addressing general principles of nutrition in T2DM (e.g., meal regularity, healthy food choices, and the impact of diet on glycemic control) as well as the source and perceived sufficiency of prior DFI-related education received from healthcare professionals (physicians, nurses, dietitians).

The questionnaire was developed based on current clinical guidelines, published literature on DFIs, and expert clinical experience in diabetes management and nutrition. Item selection was guided by commonly prescribed anti-diabetic therapies and clinically relevant DFI scenarios encountered in routine clinical practice.

Prior to administration, the instrument underwent expert content review by a pharmacist and a certified dietitian to ensure clarity, clinical relevance, and alignment with contemporary therapeutic recommendations. Minor linguistic and structural modifications were introduced following this review.

However, the questionnaire did not undergo formal psychometric validation procedures, such as assessment of internal consistency, test-retest reliability, or construct validity, nor was it pilot-tested in an independent population. As the instrument was developed within a single national healthcare context and was not standardized or cross-culturally adapted, its reproducibility and external comparability may be limited. Therefore, the resulting knowledge score should be interpreted as an exploratory measure of patient awareness rather than a validated diagnostic or predictive tool. As such, the questionnaire should be regarded as an exploratory research instrument rather than a standardized assessment tool suitable for diagnostic or interventional evaluation.

### 2.4. Statistical Analysis

Statistical analyses were performed using Statistica (data analysis software system, version 13, TIBCO Software Inc., Palo Alto, CA, USA, (2017), applying appropriate non-parametric and categorical methods due to the nature of the variables. Categorical variables were expressed as numbers and percentages.

Patient knowledge regarding DFIs was assessed using a structured questionnaire. Each correct answer was scored as 1 point, while incorrect or “I do not know” responses were scored as 0 points. A total knowledge score was calculated by summing individual items. Based on the distribution of the total knowledge score, participants were categorized into tertiles representing low, moderate, and high levels of knowledge. For multivariable analysis, the level of knowledge was dichotomized into low versus moderate-to-high knowledge. The tertile-based categorization was intended to facilitate comparative analyses in this exploratory study. In addition, complementary analyses treating the knowledge score as a continuous variable were performed and yielded consistent results.

Associations between categorical variables were assessed using the chi-square test of independence or Fisher’s exact test when applicable. Differences in total knowledge scores between two groups were analyzed using the Mann-Whitney U test, while comparisons across more than two groups were performed using the Kruskal-Wallis test.

To identify independent predictors of higher knowledge regarding DFIs, a multivariable logistic regression model was constructed. Univariable logistic regression analyses were performed prior to multivariable modeling to identify candidate predictors of higher knowledge. The dependent variable was defined as having a moderate or high level of knowledge. Independent variables entered into the model included gender, age category, educational level, duration of type 2 diabetes, use of GLP-1 RAs, and attendance at dietary consultations in the previous year (≥1 vs. none).

Results of the logistic regression analysis were presented as odds ratios (ORs) with 95% confidence intervals (CIs). A two-sided *p* value < 0.05 was considered statistically significant. All analyses were conducted on a sample of 103 participants.

## 3. Results

### 3.1. Characteristics of the Study Population

A total of 103 patients with type 2 diabetes mellitus were included in the study. The demographic and clinical characteristics of the study population are presented in Table 1. Women constituted the majority of the sample, while men accounted for 17.5% of participants. No missing data were observed for gender. Regarding age distribution, most respondents were aged between 30 and 50 years, followed by individuals aged 51–70 years. Younger participants (under 30 years of age) represented 6.8% of the study population, whereas patients aged 70 years and older accounted for 2.9%. Body mass index (BMI) analysis indicated that the majority of participants were either overweight or obese. Specifically, 47.6% of patients had a BMI in the range of 25.0–29.9 kg/m^2^, and 32.0% had a BMI ≥ 30.0 kg/m^2^. Normal body weight (BMI 18.5–24.9 kg/m^2^) was observed in 18.4% of respondents, while underweight status (BMI < 18.5 kg/m^2^) was rare. In terms of educational attainment, most participants reported higher education. Secondary education was reported by 28.2% of respondents, vocational education by 6.8%, and primary education by 1.0%. The place of residence varied among participants. A total of 39.8% lived in large cities with more than 100,000 inhabitants, 25.2% resided in medium-sized cities (20,000–100,000 inhabitants), 10.7% in small cities (up to 20,000 inhabitants), and 24.3% in rural areas. With respect to the duration of type 2 diabetes mellitus, 24.3% of participants had been diagnosed less than one year prior to the study, while 37.9% reported a disease duration of 1–5 years. A duration of 6–10 years was reported by 26.2% of respondents, and 11.7% had been living with T2DM for more than 10 years. Analysis of pharmacological treatment revealed that metformin was the most commonly used anti-diabetic medication, reported by 88 participants. GLP-1 RAs were used by 31 patients, while SGLT-2 inhibitors were reported by 12 patients. Sulfonylurea use was rare (*n* = 1), and no participants reported treatment with DPP-4 Inhibitors. A proportion of patients were concurrently using medications from two or more therapeutic classes.

### 3.2. Level of Knowledge Regarding DFIs

Patients’ self-reported knowledge regarding DFIs varied: 24.3% rated their knowledge as insufficient, 32.0% as sufficient, 27.2% as good, and 16.5% as very good. These findings indicate that a considerable proportion of patients perceive limited understanding of DFIs, underscoring the need for targeted educational interventions to improve awareness and promote safe medication practices.

The percentage of answers for individual knowledge items is presented in Table 2 and Table 3. Overall, participants demonstrated variable levels of knowledge across different domains of DFIs. The highest proportion of correct responses was observed for questions related to appropriate liquid for taking oral medications, as all patients correctly identified water as the best option for this purpose.

When asked about the impact of diet on pharmacological treatment, 71.8% of patients believed their diet positively affected drug efficacy, while 3.9% perceived a negative effect, 11.7% reported no effect, and 12.6% were unsure.

Regarding specific knowledge of DFI-related risks, 43.7% of participants recognized that high dietary fiber intake may delay drug absorption, whereas 47.6% were uncertain. Only 31.1% correctly identified tetracycline and fluoroquinolone antibiotics as particularly sensitive to interactions with dairy products, with 56.3% indicating uncertainty. Most participants (89.3%) correctly stated that the effect of food on medications depends on the drug’s properties. Knowledge gaps were also evident in relation to chronic drug use and potential nutrient deficiencies: 23.3% correctly identified vitamin B12 deficiency as a possible consequence of long-term proton pump inhibitor use, while 56.3% were unaware. Regarding grapefruit juice, only 34.0% correctly noted it may inhibit drug metabolism increasing adverse effect risk, whereas 41.7% incorrectly claimed its potential to reduce drug efficacy. Patients showed high awareness of alcohol-related risks: 99.0% correctly identified that alcohol may cause hypoglycemia, hyperglycemia, or lactic/ketoacidosis in patients taking anti-diabetic medications. Similarly, 82.5% recognized the need to monitor vitamin B12 levels during metformin therapy as a result of vitamin B12 deficiency caused by metformin. Knowledge of pharmacological mechanisms and dietary interactions varied across drug classes. For example, only 52.4% correctly identified GLP-1 agonists as drugs that can increase satiety and decrease appetite. Regarding hypoglycemia after alcohol consumption with sulfonylureas, 66.0% knew that glucose intake is recommended. Overall, patients demonstrated good practical awareness of some aspects of DFIs, such as proper administration with water, alcohol risks, and dietary fiber effects. However, significant knowledge gaps exist in areas such as specific DFIs, nutrient deficiencies, and drug metabolism modulation, indicating a need for targeted educational interventions to enhance safe medication practices.

Patients’ actual knowledge regarding DFIs was assessed using a structured questionnaire. Each correct answer (underlined in Table 2 and Table 3) was scored as 1 point and incorrect or “I do not know” answers as 0 points. A total knowledge score was calculated by summing individual items. The total knowledge score ranged from 6 to 17 points. Based on the distribution of scores, participants were categorized into tertiles representing low, moderate, and high levels of knowledge. The proportion of patients classified into the low, moderate, and high knowledge tertiles was 30.1% (*n* = 31), 32.0% (*n* = 33), and 37.9% (*n* = 39), respectively.

A comparison of knowledge levels between patients is presented in Table 4. The analysis of associations between sociodemographic and clinical variables and the level of knowledge regarding DFIs revealed a statistically significant relationship only for gender (*p* = 0.026). No significant associations were observed between knowledge level and age (*p* = 0.968), educational attainment (*p* = 0.159), or duration of type 2 diabetes mellitus (*p* = 0.571). Patients using GLP-1 RAs did not demonstrate statistically significant difference in knowledge scores compared to non-users (*p* = 0.604).

### 3.3. Sources of Information and Perceived Educational Needs

Results regarding patients’ education and counseling regarding DFIs in T2DM are presented in Table 5. The majority of patients reported not receiving any information regarding interactions between anti-diabetic medications and food (72.8%). Among those who received advice, 18.4% obtained it from a physician, 7.8% from a dietitian, and 1.0% from a nurse. Regarding dietary consultations in the past year, nearly half of the patients (49.5%) did not attend any consultations, 36.9% had 1–2 consultations, and 13.6% had 3–5 consultations; no patient reported more than five consultations. Most patients (72.8%) expressed that it is very important for their doctor or dietitian to discuss anti-diabetic DFIs more often, while 24.3% indicated that they would like such discussions only if it directly affects their treatment. Only a small proportion (2.9%) considered the topic unimportant, and none of the patients reported having sufficient knowledge to forgo such discussions. These results highlight a significant gap in patient education regarding DFIs, as well as a strong patient demand for more frequent guidance from healthcare professionals.

No statistically significant association was observed between receiving professional advice on DFIs and the level of DFI knowledge (*p* = 0.315, Table 4). Similarly, patients who reported receiving such advice did not differ significantly in total knowledge scores compared with those who had not received any counseling (*p* = 0.843, Table 4).

A statistically significant association was observed between the number of dietary consultations in the previous year and the level of knowledge regarding DFIs (*p* = 0.041, Table 4). In addition, the total knowledge score differed significantly across consultation frequency categories (*p* = 0.042, Table 4), with higher scores noted among patients who had attended at least one dietary consultation.

In the multivariable logistic regression analysis (Table 6), attending at least one dietary consultation in the previous year was independently associated with a higher level of knowledge regarding DFIs (OR = 2.31; 95% CI: 1.04–5.15; *p* = 0.039). No significant associations were observed for age, educational level, duration of diabetes, or GLP-1 RA therapy.

## 4. Discussion

The present study assessed the level of knowledge regarding DFIs among patients with T2DM, with particular focus on those receiving GLP-1 RA therapy. Our findings indicate that, despite general awareness of diabetes management, significant gaps persist in patient knowledge regarding specific DFIs, especially those relevant to modern anti-diabetic regimens. To our knowledge, this is one of the first studies to specifically evaluate patient knowledge regarding DFIs in the context of GLP-1 RA therapy, linking this knowledge directly to nutritional counseling practices rather than pharmacotherapy alone.

Therefore, the observed knowledge levels should not be interpreted as a direct proxy for effective self-management or adherence, but rather as an exploratory indicator of patient awareness of potential DFIs.

Importantly, although higher knowledge levels were associated with participation in dietary consultations, the present study does not allow conclusions regarding causality or clinical effectiveness. The cross-sectional design precludes determination of whether dietary counseling improves knowledge or whether individuals with greater health engagement are more likely to seek counseling. Moreover, knowledge acquisition does not necessarily translate into sustained behavioral change, improved adherence, or measurable metabolic benefit. Therefore, the observed associations should be interpreted as indicative of a potential educational pathway rather than confirmed clinical impact.

This observation is consistent with previous research demonstrating limited patient knowledge regarding DFIs across diverse settings and disease states. For example, a cross-sectional survey conducted in South Africa reported that although a majority of patients recognized that food can interfere with the effectiveness of medications, only a minority could identify specific interactions between commonly prescribed drugs and food items such as grapefruit juice or dairy products [17]. Similarly, in a Polish population-based study, greater awareness of DFIs did not consistently translate into comparable knowledge regarding DFIs, suggesting that DFIs represent a distinct and underrecognized challenge in patient education and clinical practice [11].

The clinical implications of insufficient DFI knowledge are not merely academic. DFIs can alter drug pharmacokinetics and pharmacodynamics, potentially reducing therapeutic efficacy or increasing the risk of adverse effects. While many reviews have highlighted the theoretical mechanisms of DFIs, such as food-induced changes in drug absorption or metabolism, there is limited patient-focused research evaluating how well individuals understand these phenomena in real-world contexts for chronic conditions like diabetes. For instance, review studies emphasize that patients with chronic polypharmacy—a common scenario in T2DM—have increased risk of drug interactions overall, including food-related effects, yet awareness remains low [18].

Our study also extends previous findings by suggesting that knowledge regarding DFIs may differ according to treatment type. Patients on GLP-1 RA therapy showed no significant differences in knowledge levels compared to others, despite the unique nutritional implications of these agents. GLP-1 RAs have transformed the clinical management of T2DM and obesity and are associated with gastrointestinal side effects (e.g., nausea, constipation) that require tailored nutritional guidance to support adherence and optimize outcomes [16,19]. Although most previous studies on DFI awareness have not been stratified by specific drug classes, the general pattern of knowledge gaps supports our findings and highlights the need for therapy-specific educational strategies.

Healthcare professionals play a crucial role in bridging knowledge gaps related to DFIs; however, the present findings indicate that not all forms of counseling are equally effective. While general or opportunistic advice regarding DFIs may increase awareness, structured and nutrition-focused education appears to be more strongly associated with higher levels of patient knowledge. For instance, a pilot study among Polish physicians found that formal training improved knowledge scores on DFIs from moderate to substantially higher levels, indicating that targeted education can enhance professional competencies and, by extension, patient guidance [20]. Moreover, broader population studies on DFI awareness demonstrate demographic influences on knowledge, including associations with education level and use of reliable information sources, which are similar to patterns observed in general drug interaction research [21]. Hence, a large survey in Jordan found that higher education and healthcare exposure correlated with better understanding of DFIs, supporting the need for tailored educational programs that consider sociodemographic variables [22].

The present study provides a comprehensive assessment of factors associated with patients’ knowledge regarding DFIs in T2DM. Despite the relatively long disease duration and frequent pharmacological treatment, knowledge gaps remained substantial and were not uniformly explained by demographic or clinical characteristics.

Among the sociodemographic variables analyzed, only gender was significantly associated with the level of DFI knowledge, suggesting potential differences in health information-seeking behaviors or engagement with educational content between men and women. In contrast, age, educational attainment, and duration of diabetes were not significantly related to knowledge level. These findings indicate that longer exposure to the healthcare system or higher formal education does not necessarily translate into better understanding of DFIs, underscoring systemic shortcomings in patient education. This finding should be interpreted with caution due to the marked predominance of female participants in the study sample, which may reflect differences in health information-seeking behavior rather than true sex-related differences in knowledge.

Notably, neither the use of GLP-1 RA nor the mere receipt of advice regarding DFIs was associated with higher knowledge scores. This suggests that opportunistic or non-structured counseling may be insufficient to produce meaningful improvements in patient understanding. Similar observations have been reported in previous studies, where passive information delivery failed to improve medication-related knowledge or adherence [17].

In contrast, the number of dietary consultations emerged as a key determinant of DFI knowledge. Patients who had attended at least one dietary consultation demonstrated significantly higher knowledge levels, both in univariate analyses and in the multivariable logistic regression model. Importantly, dietary consultation remained an independent predictor of higher knowledge after adjustment for demographic and clinical factors, more than doubling the odds of achieving a moderate-to-high knowledge level. This highlights the critical role of structured, nutrition-focused education delivered by qualified professionals.

Although the study was conducted within the thematic framework of GLP-1-based therapy, the observed associations between dietary consultations and DFI knowledge were not specific to GLP-1 RA users. No statistically significant differences in knowledge were identified between patients treated with GLP-1 RAs and those receiving other anti-diabetic therapies. Therefore, the GLP-1–related component of this study should be considered exploratory and intended to stimulate further research into therapy-specific nutritional education needs rather than to establish GLP-1–specific causal pathways.

Taken together, these findings emphasize that effective education on DFIs should not be assumed to occur naturally over time or through routine clinical care alone. Instead, the results underscore the importance of interdisciplinary educational initiatives that integrate pharmacological and nutritional counseling for patients with T2DM, particularly those treated with complex regimens such as GLP-1 RAs. Integrating structured, dietitian-led education into standard diabetes care may represent a plausible strategy to address persistent knowledge gaps and strengthen patient awareness of medication-related considerations.

### Limitations

This study has several limitations that should be acknowledged. First, the cross-sectional design precludes causal inference between patient characteristics and the level of knowledge regarding DFIs.

Second, the assessment relied exclusively on a self-administered questionnaire evaluating cognitive knowledge without objective assessment of actual dietary behaviors or nutrient intake (e.g., frequency and quantity of fiber-rich foods, dairy products, or grapefruit juice). Consequently, a potential discrepancy between knowledge and real-life dietary practices could not be explored.

Third, no biochemical markers, such as glycated hemoglobin (HbA1c), vitamin B12 concentrations, or other metabolic indicators, were collected. This prevented evaluation of potential associations between knowledge of DFIs and clinical or metabolic outcomes. Future studies should integrate biochemical and dietary data to better assess the clinical relevance of patient knowledge.

Fourth, the study sample exhibited a marked gender imbalance, with a predominance of female participants. Although female gender was associated with higher knowledge in univariate analysis, it was not an independent predictor in multivariable modeling. This imbalance may reflect gender-specific health-seeking behaviors and limits the generalizability of sex-based comparisons.

Finally, the questionnaire was developed specifically for this study and underwent expert content review but did not undergo formal psychometric validation, including analyses of internal consistency, test–retest reliability, or construct validity. The absence of standardization limits reproducibility, comparability with other studies, and external validity. Furthermore, the instrument was developed within a single national healthcare context and was not cross-culturally adapted. As such, the findings may not be generalizable to other healthcare systems or cultural settings. Additionally, although GLP-1–related items were included, the study was not designed to assess therapy-specific behavioral adaptation or clinical outcomes among GLP-1 RA users. Accordingly, the results should be interpreted as exploratory and hypothesis-generating.

Additionally, although multivariable adjustment was performed, residual confounding cannot be excluded. Unmeasured factors such as general health literacy, intensity of healthcare engagement, or intrinsic motivation for self-education may have influenced the observed associations.

## 5. Conclusions

This cross-sectional study demonstrates that patients with T2DM exhibit substantial gaps in knowledge regarding DFIs, despite ongoing pharmacological treatment. Participation in at least one dietary consultation was independently associated with higher knowledge levels; however, no causal inference can be drawn regarding the direction of this association.

The findings indicate that exposure to healthcare services or advanced pharmacotherapy, including GLP-1 receptor agonists, does not automatically ensure adequate awareness of clinically relevant DFIs. At the same time, the study assessed declarative knowledge rather than actual dietary behavior, treatment adherence, or metabolic control. Therefore, improved knowledge cannot be assumed to translate into improved clinical outcomes.

From a clinical systems perspective, structured nutritional counseling may represent a plausible pathway to strengthen patient education regarding DFIs. Nevertheless, longitudinal and interventional research is required to determine whether enhanced knowledge leads to measurable improvements in safety, adherence, or glycemic control. The present findings should therefore be considered exploratory and hypothesis-generating.

## Figures and Tables

**Table 1:** Characteristics of the study population.

Variable		*n* = 103	%
gender	female	85	82.5
	male	18	17.5
	undefined	0	0
age	<30 years	7	6.8
	30–50 years	61	59.2
	51–70 years	28	27.2
	70 years	3	2.9
BMI (kg/m^2^)	<18.5	2	1.9
	18.5–24.9	19	18.4
	25.0–29.9	49	47.6
	>30.0	33	32.0
highest level of education	primary	1	1.0
	vocational	7	6.8
	secondary	29	28.2
	higher	66	64.1
place of residence	village	25	24.3
	small city (up to 20,000 inhabitants)	11	10.7
	medium city (20,000–100,000 inhabitants)	26	25.2
	large city (over 100,000 inhabitants)	41	39.8
lasting of T2DM	<1 year	25	24.3
	1–5 years	39	37.9
	6–10 years	27	26.2
	>10 years	12	11.7
type of medications *	metformin	88	
	sulfonylureas	1	
	DPP-4 Inhibitors	0	
	GLP-1 Receptor Agonists	31	
	SGLT-2 Inhibitors	12	

* some of patients were concurrently using medications from two or more therapeutic classes. Abbreviations: BMI-body mass index; T2DM—type 2 diabetes mellitus; DPP-4 inhibitors—dipeptidyl peptidase-4 inhibitors; GLP-1 receptor agonists—glucagon-like peptide-1 receptor agonist; SGLT-2 inhibitors—sodium-glucose cotransporter 2 inhibitors.

**Table 2:** General DFI knowledge and diabetes-related items.

		*n* = 103	%
How do you assess your level of knowledge about drug–food interactions?	Very good	17	16.5
	Good	28	27.2
	Sufficient	33	32.0
	Insufficient	25	24.3
How does your diet affect the pharmacological treatment you are using?	It has a positive effect, increasing its effectiveness.	74	71.8
	It has a negative effect, decreasing its effectiveness.	4	3.9
	It does not affect its effectiveness in any way.	12	11.7
	I don’t know.	13	12.6
What is the best way to wash down orally administered medications?	Tea	0	0.0
	Juice	0	0.0
	Water	103	100.0
	I don’t know	0	0.0
What is the potential risk of consuming large amounts of dietary fiber while taking medications?	It may delay their absorption.	45	43.7
	It may increase their concentration in the blood.	2	1.9
	It may cause hypoglycemia.	7	6.8
	I don’t know.	49	47.6
Which group of drugs is particularly sensitive to interactions with dairy products?	Anticoagulant drugs	8	7.8
	Beta-blockers	5	4.9
	Tetracycline and fluoroquinolone antibiotics	32	31.1
	I don’t know.	58	56.3
How can food consumption affect the effectiveness and safety of taking medications?	All medications should be taken on an empty stomach to ensure maximum absorption.	2	1.9
	All medications should be taken during a meal to avoid stomach irritation.	7	6.8
	The method of taking the drug depends on its properties-some drugs should be taken on an empty stomach, others with a meal to ensure optimal effect.	92	89.3
	Food consumption does not affect the action of medications because the body absorbs them in the same way regardless of the time of administration.	2	1.9
The result of chronic use of proton pump inhibitors (PPIs), such as Omeprazole or Lansoprazole, may be:	Vitamin B12 deficiency	24	23.3
	Fatty diarrhea	6	5.8
	Calcium deficiency resulting from reduced absorption	15	14.6
	I don’t know.	58	56.3
Why should you avoid swallowing down medications with grapefruit juice?	It may accelerate the action of the drugs.	10	9.7
	It may inhibit the metabolism of some drugs, increasing the risk of adverse effects.	35	34.0
	It may reduce the effectiveness of the drugs.	43	41.7
	I don’t know.	15	14.6
Why should patients using anti-diabetic medications be cautious about consuming alcohol?	Alcohol may increase the risk of hypoglycemia or hyperglycemia, as well as lactic acidosis (with Metformin) or ketoacidosis (with SGLT-2 inhibitors).	102	99.0
	Alcohol consumption causes the immediate elimination of medications from the body.	1	1.0
	Alcohol always increases the effectiveness of anti-diabetic drugs.	0	0.0
	Alcohol has no effect on diabetes treatment.	0	0.0
Why should a patient taking Metformin regularly monitor their Vitamin B12 level?	Because Metformin may lead to its deficiency.	85	82.5
	Because Metformin causes excessive absorption of Vitamin B12.	6	5.8
	Because Vitamin B12 neutralizes the effect of Metformin.	4	3.9
	Because Vitamin B12 reduces the risk of lactic acidosis.	8	7.8
A patient taking Sulfonylureas consumed a large amount of alcohol. What should they do in case of hypoglycemic symptoms?	Drink a glass of Coca-Cola or consume glucose.	68	66.0
	Take an additional dose of the drug.	2	1.9
	Fast until the next dose.	6	5.8
	Consume a large amount of fats to reduce the effect of alcohol.	27	26.2
Which of the following side effects may occur as a result of an interaction between grapefruit juice and Sulfonylureas?	Hyperglycemia	15	14.6
	Hypoglycemia	31	30.1
	Lactic acidosis	45	43.7
	Hypertension	12	11.7
How does tobacco affect the action of medications used in Type 2 Diabetes?	It accelerates the metabolism of Metformin.	5	4.9
	It may reduce the effectiveness of Insulin and oral medications.	60	58.3
	It causes hypoglycemia.	3	2.9
	It does not affect diabetes treatment.	35	34.0
Which of the following food products can improve Vitamin B12 absorption?	Wholemeal bread	13	12.6
	Citrus fruits	9	8.7
	Meat, eggs, dairy	61	59.2
	Dark chocolate	20	19.4
Why is a diet rich in dietary fiber beneficial for people with Type 2 Diabetes?	It helps in better control of blood glucose levels.	70	68.0
	It increases appetite and helps digest sugars faster.	8	7.8
	It lowers blood sugar by increasing insulin secretion.	25	24.3
	It causes rapid blood sugar spikes.	0	0.0
What is the importance of meal regularity in Type 2 Diabetes?	It helps maintain stable blood glucose levels.	100	97.1
	It has no effect on blood sugar levels.	2	1.9
	It may lead to hypoglycemia.	0	0.0
	It causes excessive insulin secretion.	1	1.0
Which of the following drinks is the best choice for a person with diabetes?	Sweetened carbonated drinks	0	0.0
	100% fruit juices	1	1.0
	Mineral water	102	99.0
	Coffee with sugar and milk	0	0.0
What is the best source of protein for people with Type 2 Diabetes?	Red meat	11	10.7
	Plant-based proteins (e.g., lentils, chickpeas) and lean meat, fish	92	89.3
	Processed cold cuts	0	0.0
	Fast food, e.g., hamburgers	0	0.0

For each questionnaire item, the correct answer is underlined. Abbreviations: DPP-4 inhibitors—dipeptidyl peptidase-4 inhibitors; SGLT-2 inhibitors—sodium-glucose co-transporter 2 inhibitors.

**Table 3:** GLP-1 RA-specific items.

		*n* = 103	%
Which group of medications can cause increased satiety and decreased appetite?	DPP-4 Inhibitors	16	15.5
	Sulfonylureas	13	12.6
	GLP-1 Receptor Agonists	54	52.4
	SGLT-2 Inhibitors	20	19.4
How can coffee affect the action of GLP-1 Agonists (e.g., Ozempic, Victoza)?	It can cause stronger nausea and gastrointestinal disturbances.	55	53.4
	It improves the effectiveness of these drugs.	1	1.0
	It reduces the absorption of the drugs.	12	11.7
	It has no effect on the action of GLP-1 receptor agonists.	35	34.0

For each questionnaire item, the correct answer is underlined.

**Table 4:** Factors associated with the level of knowledge regarding DFIs.

Variable	Statistical Test	Outcome	*p* Value
Gender	χ^2^ test	Significant association with knowledge level	**0.026**
Age category	χ^2^ test	No significant association	0.968
Educational level	χ^2^ test	No significant association	0.159
Duration of T2DM	χ^2^ test	No significant association	0.571
Use of GLP-1 RA	Mann–Whitney U	No difference in knowledge score	0.604
Receiving advice on DFIs (yes/no)	χ^2^ test	No association with knowledge level	0.315
	Mann–Whitney U	No difference in total knowledge score	0.843
Number of dietary consultations (categories)	χ^2^ test	Significant association with knowledge level	**0.041**
	Kruskal–Wallis	Significant difference in knowledge score	**0.042**
≥1 dietary consultation (multivariable model)	Logistic regression	Independent predictor of higher knowledge (OR 2.31, 95% CI 1.04–5.15)	**0.039**

Boldface *p*-values denote statistically significant associations (*p* < 0.05). Abbreviations: DFIs—drug–food interaction; OR—odds ratio; CI—confidence interval; T2DM—type 2 diabetes mellitus; GLP-1 RA—glucagon-like peptide-1 receptor agonist.

**Table 5:** Patient education and counseling regarding DFIs in T2DM.

		*n* = 103	%
Advice regarding interactions between anti-diabetic medications and food	Yes, from a physician	19	18.4
	Yes, from a dietitian	8	7.8
	Yes, from a nurse	1	1.0
	No, I have never received such information	75	72.8
Number of dietary consultations in the last year	0	51	49.5
	1–2	38	36.9
	3–5	14	13.6
	>5	0	0.0
Would you like your doctor or dietitian to discuss the topic of anti-diabetic drug–food interactions more often?	Yes, it is very important.	75	72.8
	Yes, but only if it affects my treatment.	25	24.3
	No, I already have sufficient knowledge.	0	0.0
	No, it is not important to me.	3	2.9

Abbreviations: T2DM—type 2 diabetes mellitus; DFIs—drug–food interactions.

**Table 6:** Univariable and multivariable logistic regression analysis of factors associated with higher knowledge of DFIs.

Variable	OR	95% CI	*p* Value
Female gender	1.74	0.78–3.88	0.170
Age (categories)	1.03	0.81–1.31	0.782
Higher education	1.42	0.61–3.29	0.410
Duration of T2DM	1.08	0.86–1.36	0.490
Use of GLP-1 RAs	0.94	0.43–2.07	0.880
≥1 dietary consultation in the previous year	**2.31**	**1.04–5.15**	**0.039**

Dependent variable: moderate-to-high level of knowledge regarding drug–food interactions (vs. low level). Bold *p*-values denote statistically significant predictors in the regression model (*p* < 0.05). Abbreviations: DFIs—drug–food interactions; T2DM—type 2 diabetes mellitus; GLP-1 RA—glucagon-like peptide-1 receptor agonist; OR—odds ratio; CI—confidence interval.

## Data Availability

Data available on request from the corresponding author.

## Supplementary Materials

The following supporting information can be downloaded at: https://www.mdpi.com/article/10.3390/nu18050742/s1, Survey S1.
