# Supplementary material for: Nutritional Counseling Is Independently Associated with Greater Knowledge of Drug–Food Interactions in Patients with Type 2 Diabetes
Source: Nutrients. 2026 Feb 26;18(5):742. doi: 10.3390/nu18050742 (PMC12987031; doi:10.3390/nu18050742)
Supplement: Supplementary file 1 [file nutrients-18-00742-s001.zip › nutrients-4087994-supplementary.pdf]

Title: Assessment of Patient Knowledge of Drug–Food Interactions in Type 2 Diabetes: Implications for Nutritional Support of GLP-1 Receptor Agonist Therapy.

Introduction to the Respondent

Dear Sir or Madam,

You are invited to participate in a scientific study concerning the knowledge of patients with Type 2 Diabetes Mellitus (T2DM) regarding the influence of food on the effectiveness of pharmacological treatment and drug–food interactions. This study is part of my Master's thesis conducted at the University of Economics and Human Sciences in Warsaw.

The questionnaire you are about to complete covers the following issues:

Knowledge about the influence of diet on the treatment of Type 2 Diabetes.

Awareness of food interactions with medications used in Type 2 Diabetes therapy.

Dietary habits.

The estimated time to complete the survey is approximately 15-20 minutes.

The study is scientific in nature, and its results will be used exclusively for academic purposes, including the preparation of my Master's thesis. All data will be presented in aggregate form, without the possibility of identifying individual persons.

The aim of the study is to assess the level of knowledge of patients with Type 2 Diabetes regarding the influence and interactions of food on the effectiveness of pharmacological treatment.

Participation in the study is entirely voluntary and anonymous. You may withdraw from the study at any time, without giving a reason and without any consequences. If you have any questions or concerns, please contact me (korbej4317425_aeh@students.vizja.pl).

Thank you for your time and participation in the study!

Sincerely,

Joanna Korbela.

Part I: General Information

1. Gender:

   a) Female
   b) Male
   c) Undefined

2. Age (in years):

   a) <30 years
   b) 30-50 years
   c) 51-70 years
   d) >70 years

3. Highest Level of Education:

   a) Primary
   b) Vocational
   c) Secondary
   d) Higher (University)

4. Place of Residence:

a) Village
b) Small city (up to 20,000 inhabitants)
c) Medium city (20,000 - 100,000 inhabitants)
d) Large city (over 100,000 inhabitants)

5. How long have you had Type 2 Diabetes?

a) Less than 1 year
b) 1-5 years
c) 6-10 years
d) More than 10 years

6. Which medications do you use to treat Type 2 Diabetes? (You can select more than one answer):

Metformin (e.g., Avamina, Glucophage, Siofor)
Sulfonylureas (e.g., Glimepiride, Glipizide)
DPP-4 Inhibitors (e.g., Sitagliptin, Linagliptin)
GLP-1 Agonists (e.g., Victoza, Saxenda, Ozempic, Trulicity)
SGLT-2 Inhibitors - Flozins (e.g., Forxiga, Jardiance, Invokana)

7. What is your Body Mass Index (BMI)?

a) Underweight (BMI < 18.5)
b) Normal weight (BMI 18.5 - 24.9)
c) Overweight (BMI 25.0 - 29.9)
d) Obesity (BMI > 30.0)

Part II: Knowledge of Drug–Food Interactions (DFIs)

8. How do you assess your level of knowledge about drug–food interactions?

a) Very good
b) Good
c) Sufficient
d) Insufficient

9. How does your diet affect the pharmacological treatment you are using?

a) It has a positive effect, increasing its effectiveness.
b) It has a negative effect, decreasing its effectiveness.
c) It does not affect its effectiveness in any way.
d) I don't know.

10. What is the best way to wash down orally administered medications?

a) Tea
b) Juice
c) Water
d) I don't know

11. What is the potential risk of consuming large amounts of dietary fiber while taking medications?

a) It may delay their absorption.
b) It may increase their concentration in the blood.
c) It may cause hypoglycemia.
d) I don't know.

12. Which group of drugs is particularly sensitive to interactions with dairy products?

a) Anticoagulant drugs
b) Beta-blockers
c) Tetracycline and fluoroquinolone antibiotics
d) I don't know.

13. How can food consumption affect the effectiveness and safety of taking medications?

a) All medications should be taken on an empty stomach to ensure maximum absorption.
b) All medications should be taken during a meal to avoid stomach irritation.
c) The method of taking the drug depends on its properties - some drugs should be taken on an empty stomach, others with a meal to ensure optimal effect.
d) Food consumption does not affect the action of medications because the body absorbs them in the same way regardless of the time of administration.

14. The result of chronic use of proton pump inhibitors (PPIs), such as Omeprazole or Lansoprazole, may be:
    a) Vitamin B12 deficiency
    b) Fatty diarrhea
    c) Calcium deficiency resulting from reduced absorption
    d) I don't know.
15. Why should you avoid swallowing down medications with grapefruit juice?
    a) It may accelerate the action of the drugs.
    b) It may inhibit the metabolism of some drugs, increasing the risk of adverse effects.
    c) It may reduce the effectiveness of the drugs.
    d) I don't know.
16. Why should patients using anti-diabetic medications be cautious about consuming alcohol?
    a) Alcohol may increase the risk of hypoglycemia or hyperglycemia, as well as lactic acidosis (with Metformin) or ketoacidosis (with SGLT-2 Inhibitors).
    b) Alcohol consumption causes the immediate elimination of medications from the body.
    c) Alcohol always increases the effectiveness of anti-diabetic drugs.
    d) Alcohol has no effect on diabetes treatment.
17. Why should a patient taking Metformin regularly monitor their Vitamin B12 level?
    a) Because Metformin may lead to its deficiency.
    b) Because Metformin causes excessive absorption of Vitamin B12.
    c) Because Vitamin B12 neutralizes the effect of Metformin.
    d) Because Vitamin B12 reduces the risk of lactic acidosis.
18. Which group of medications can cause increased satiety and decreased appetite?
    a) DPP-4 Inhibitors
    b) Sulfonylureas
    c) GLP-1 Agonists
    d) SGLT-2 Inhibitors
19. A patient taking Sulfonylureas consumed a large amount of alcohol. What should they do in case of hypoglycemic symptoms?
    a) Drink a glass of Coca-Cola or consume glucose.
    b) Take an additional dose of the drug.
    c) Fast until the next dose.
    d) Consume a large amount of fats to reduce the effect of alcohol.
20. How can coffee affect the action of GLP-1 Agonists (e.g., Ozempic, Victoza)?
    a) It can cause stronger nausea and gastrointestinal disturbances.
    b) It improves the effectiveness of these drugs.
    c) It reduces the absorption of the drugs.

d) It has no effect on the action of GLP-1 Agonists.

21. Which of the following side effects may occur as a result of an interaction between grapefruit juice and Sulfonylureas?
    a) Hyperglycemia
    b) Hypoglycemia
    c) Lactic acidosis
    d) Hypertension
22. How does tobacco affect the action of medications used in Type 2 Diabetes?
    a) It accelerates the metabolism of Metformin.
    b) It may reduce the effectiveness of Insulin and oral medications.
    c) It causes hypoglycemia.
    d) It does not affect diabetes treatment.

Part III: Knowledge of Diet and Diabetes Education

23. Which of the following food products can improve Vitamin B12 absorption?
    a) Wholemeal bread
    b) Citrus fruits
    c) Meat, eggs, dairy
    d) Dark chocolate
24. Why is a diet rich in dietary fiber beneficial for people with Type 2 Diabetes?
    a) It helps in better control of blood glucose levels.
    b) It increases appetite and helps digest sugars faster.
    c) It lowers blood sugar by increasing insulin secretion.
    d) It causes rapid blood sugar spikes.
25. What is the importance of meal regularity in Type 2 Diabetes?
    a) It helps maintain stable blood glucose levels.
    b) It has no effect on blood sugar levels.
    c) It may lead to hypoglycemia.
    d) It causes excessive insulin secretion.
26. Which of the following drinks is the best choice for a person with diabetes?
    a) Sweetened carbonated drinks
    b) 100% fruit juices
    c) Mineral water
    d) Coffee with sugar and milk
27. What is the best source of protein for people with Type 2 Diabetes?
    a) Red meat
    b) Plant-based proteins (e.g., lentils, chickpeas) and lean meat, fish
    c) Processed cold cuts
    d) Fast food, e.g., hamburgers
28. Have you received advice regarding interactions between anti-diabetic medications and food?
    a) Yes, from a physician
    b) Yes, from a dietitian
    c) Yes, from a nurse
    d) No, I have never received such information
29. How many times have you had dietary consultations in the last year?
    a) 0 times
    b) 1-2 times
    c) 3-5 times
    d) More than 5 times

30. Would you like your doctor or dietitian to discuss the topic of anti-diabetic drug–food interactions more often?
    a) Yes, it is very important.
    b) Yes, but only if it affects my treatment.
    c) No, I already have sufficient knowledge.
    d) No, it is not important to me.
